# Transcutaneous Electrical Acupoint Stimulation Accelerates the Recovery of Gastrointestinal Function after Cesarean Section: A Randomized Controlled Trial

**DOI:** 10.1155/2018/7341920

**Published:** 2018-11-13

**Authors:** Dandan Zhou, Bo Hu, Shan He, Xiaogang Li, Hui Gong, Feng Li, Qiang Wang

**Affiliations:** ^1^Department of Anesthesiology, Xijing Hospital, Fourth Military Medical University, Xi'an 710032, Shaanxi Province, China; ^2^Department of Anesthesiology, The Northwest Women's and Children's Hospital, Xi'an 710061, Shaanxi Province, China; ^3^Department of Anesthesiology, The First Affiliated Hospital of Xi'an Jiaotong University, Xi'an 710061, Shaanxi Province, China

## Abstract

**Background:**

Gastrointestinal functional recovery is an important factor affecting postoperative outcome. The aim of this study was to evaluate the effect of transcutaneous electrical acupoint stimulation (TEAS) on gastrointestinal function in women undergoing cesarean section.

**Methods:**

150 pregnant women undergoing cesarean section were randomly allocated into TEAS, nonacupoint stimulation (sham group), and no stimulation (control group). The primary outcome was indications of gastrointestinal functional recovery and the secondary outcomes included time to first mobilization, postoperative hospital stay, daily living activities at one week after surgery, postoperative side-effects, and serum levels of gastroenterological hormones.

**Results:**

The time to first flatus in TEAS group was significantly shorter compared to control (*P*=0.004) and sham groups (*P*=0.003). The time to first oral liquid and solid intake was significantly shorter than that in control (*P*<0.001;* P*=0.021) and sham group (*P*=0.019;* P*=0.037). Besides, postoperative hospital stay was shorter in TEAS group than in control group (*P*=0.031) and sham group (*P*<0.001). TEAS also promoted daily living activities (*P*=0.001 versus control group and* P*=0.015 versus sham group). Postoperative complications were similar among all the groups except for the incidence of abdominal distention 24 h after surgery (*P*=0.013;* P*=0.040). The motilin level was increased by TEAS (*P*=0.014 versus control group and* P*=0.020 versus sham group).

**Conclusion:**

TEAS accelerated gastrointestinal functional recovery after cesarean section, by reducing postoperative hospital length, and improved daily living activities after surgery. This effect was partially mediated by regulation of the gastroenterological hormones.

## 1. Background

Cesarean section or lower uterine segment section is one of the most common abdominal surgical procedures used today. According to the World Health Organization, the rate of cesarean section was 46% in China and 5% ~20% Globally [1]. Between 2008 and 2014, the overall annual rate of cesarean deliveries in China was 34.9% [2]. After cesarean section, women undergo a delayed return of gastrointestinal function, which is defined as postoperative ileus (POI), which is still a problematic and frequent complication after surgery [3]. This consecutively increases hospital stay, postoperative pain, abdominal distension, and inability to breastfeed, which eventually delays the recovery. The incidence of mild ileus has a mean incidence of 10% (range of 2-21%) after cesarean section [4, 5]. Therefore, improving the rehabilitation of gastrointestinal function after cesarean section is crucial during the perioperative period.

Acupuncture is a form of traditional Chinese medicine which is widely practiced in China and has been used in the treatment of a variety of digestive system diseases [6]. Previous studies have demonstrated that acupuncture had positive effects on functional dyspepsia, functional constipation, inflammatory diarrhea, and postoperative paralytic ileus [7–10]. Due to the positive effects of acupuncture, the studies are utilizing acupuncture for treating gastrointestinal diseases and hence enormous attention has been paid. Despite data that support the role of postoperative acupuncture in the gastrointestinal functional recovery after colorectal surgery [11], the data supporting the practice of acupuncture in cesarean section are quite limited. Currently, many research studies have adopted the noninvasive transcutaneous electrical acupoint stimulation, which is a modified form of acupuncture. This was proved to be as effective as acupuncture and electroacupuncture [12]. Hence, in this study TEAS was used to investigate its effect on gastrointestinal functional recovery in women undergoing cesarean section.

## 2. Methods

This is a 3-site, prospective, randomized, controlled trial study conducted in the Northwest Women's and Children's Hospital in China from September 2015 to March 2016. The study followed declaration of Helsinki and was approved by the Medicine Research Ethics Committee of China Northwest Women's and Children's Hospital. The trial was registered at clinicaltrials.gov (NCT02416310). The information of the study was explained to all enrolled participants, and written informed consent was obtained from each participant.

### 2.1. Study Population

Participants who were aged 18 years or over, undergoing selective cesarean section with American Society of Anesthesiologists grade I&II, were recruited. Exclusion criteria were emergency operation, previous bowel surgery, chronic digestive disorders, and presence of diabetes or hypertension. Moreover, participants with TEAS contraindications, such as the broken skin of the related points, local tumor, and installed pacemakers, were also excluded. Patients were excluded from analysis for severe postpartum hemorrhage and postoperative admission to intensive care unit for any reason.

### 2.2. Sample Size Calculation

For primary outcome measurements, we calculated that a sample of 150 participants (50 per group) was required in order to detect a decrease in the time to first passage of flatus, which was from 30 h (an average first flatus time from a pilot study) to 21 h. The study was designed to have a Type I error of 0.05, a power of 80%, and loss to follow-up of 15%.

### 2.3. Randomization and Blinding

The participants were randomly allocated to the study groups using a computerized random number generation program by PASS 11.0. Allocation concealment was enclosed in sealed, opaque, sequentially numbered envelopes which will be opened only upon arrival of the participant in the operation room. 150 pregnant women undergoing cesarean section were randomly allocated into three groups (50 subjects in each group): TEAS, nonacupoint stimulation group (sham group), and no stimulation (control group). Randomization was implemented by the research designer, who did not participate in recruitment and data collection. The research participants were screened according to the inclusion and exclusion criteria. All study data were collected after the interventions. All subjects and research staff involved in recruitment, data collection, and statistical analysis were blinded to the randomization status throughout the study. Only the acupuncturist knew about the interventions the subjects received, but he was not involved in the research process, except for the implementation of TEAS. He was not involved in data collection and was asked to maintain the secrecy about the interventions he provided.

All participants were preoperatively fasted for at least 8 h without intake of solids and 2 h without intake of clear fluids. All surgeries were performed according to a standard protocol. Demographic and clinical data including age, gestational age, body mass index (BMI), obstetrics conditions, and underlying medical were collected after enrollment.

### 2.4. TEAS Protocol

**Figure 1:**
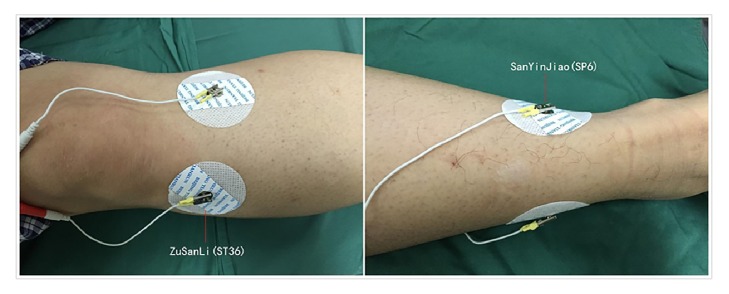
The acupoints used in this study. Bilateral Sanyinjiao (SP6) and Zusanli (ST36) were chosen as the acupuncture points. ST36, three cun below the lower border of patella, one cun lateral to the anterior crest of the tibia, in the tibialis anterior muscle. SP6, three cun directly above the tip of the medial malleolus, on the posterior border of the medical aspect of the tibia. According to the theory of traditional Chinese medicine, one cun is the distance between the two ends of the creases of the proximal and distal interphalangeal joints of the subject's index finger when flexed.

TEAS was performed twice by an experienced acupuncturist. The first electrical stimulation was administered 30 min before surgery and the second electrical stimulation was administered 6 h after surgery. Bilateral Zusanli (ST36) and Sanyinjiao (SP6) were chosen as the acupuncture points according to the theory of traditional Chinese medicine. The location of acupoints was illustrated in Figure 1. TEAS was performed using cutaneous electrode pads (5 × 5 mm^2^) which were placed bilaterally at SP6 and ST36 acupoints and were connected with Hwato electric acupuncture treatment instrument (model No. SDZ-V; Suzhou Medical Appliances Co. Ltd, Suzhou, China). Participants in the TEAS group received electrical stimulation with a dilatational wave of 2/10 Hz (2-second cycle) for 30 min. The intensity was gradually increased to 7-11 mA to achieve a satisfactory De-Qi sensation of heaviness, numbness, and swelling. Participants in the sham group received electrical stimulation at a nonacupoint which was located 2 cm interior to the ST36 and SP6. Participants in the control group only had electrode pads applied, but did not give electrical stimulation. The second electrical stimulation was administered to participants 6 h after surgery just the same as the first program.

### 2.5. Anesthesia and Perioperative Care

All the anesthesia procedures were conducted according to a standard combined-spinal epidural anesthesia with standard monitoring. The combined-spinal epidural anesthesia procedure was performed with a needle (27G) through needle (18G) technique. After free flow of cerebrospinal fluid, 20 *μ*g of fentanyl plus 10 mg of 0.5% ropivacaine hydrochloride was injected for over 15 seconds, and an epidural catheter was in place and used for postoperative epidural patient-controlled analgesia. The operating table was adjusted to make sure that the sensory block reached T_4_ when surgery started. 2% lidocaine was administered by the epidural catheter when needed. All surgeries were conducted according to a standard protocol. Hypotension (fall in systolic blood pressure >20% from baseline) and bradycardia (with heart rate <55 beats min^−1^) were treated with 10 mg ephedrine and 0.5mg atropine through intravenous bolus injection, respectively. The conditions of nausea and vomiting during surgery were treated with ondansetron. Dexamethasone was given intravenously when there is possible nerve injury during anesthesia.

After surgery, participants can decide whether or not to receive postoperative analgesia. A standard epidural or intravenous patient-controlled analgesia was given in accordance with their wishes. Management after surgery was also standardized; participants who did not experience postoperative bloating, nausea, and vomiting were instructed to consume a liquid diet, and normal diet was given after first flatus.

### 2.6. Data Collection

Pulse oximetry, electrocardiogram, and noninvasive blood pressure were recorded at 3 min intervals until the completion of surgery. The following parameters were monitored and recorded during surgery: duration of surgery, blood loss during surgery, total intraoperative infusion, and intraoperative medication usage (ondansetron, dexamethasone, ephedrine, and atropine). Before the first electrical stimulation and after the second electrical stimulation, 3 ml venous blood of all participants was obtained to detect serum gastroenterological hormones.

Participants were followed up every day until being discharged. The time of first flatus passage and defecation, first liquid and normal diet intake after surgery, postoperative side-effects (severity of nausea/vomiting in 24 h after surgery, incidences of abdominal distention and POI), postoperative hospital stay, activities of daily living at one week after surgery were recorded. Activities of daily living at one week were obtained by telephone follow-up if the patient had been discharged. In addition, the postoperative pain at rest and with movement at 6 h, 24 h, and 48 h after surgery was also recorded using numeric rating scale (NRS).

### 2.7. Outcomes and Definition

The primary outcome was the time to first passage of flatus. The secondary outcomes included the time to first passage of defecation, the first time to oral liquid and normal diet intake, the first time to mobilization, postoperative side-effects (abdominal distention, POI, nausea, and vomiting), postoperative hospital stay, postoperative pain, and performance in the activities of daily living at one week after surgery. Postoperative hospital stay was defined as number of hours stay in hospital after surgery including the day of surgery and the day of discharge. Participants were discharged when their wound healed well and they were able to tolerate nutrition, pass flatus or defecate, and perform regular daily activities with or without home care. The performance in activities of daily living was measured using Barthel index [13]. The severity of nausea and vomiting was assessed by verbal descriptive scale (VDS) (VDS 0, no nausea and vomiting; VDS 1, nausea but no need for antiemetic treatment; VDS 2, nausea and need for antiemetic treatment; VDS 3, nausea with retching or vomiting) [14]. Abdominal distention was defined as belching, perception of fullness, bowel sound, and difference of abdominal girth. POI was considered to be present when two or more of the following criteria were met: (a) nausea or vomiting; (b) inability to tolerate an oral diet over last 24 h; (c) absence of flatus over last 24 h; (d) abdominal distension; and (e) radiologic confirmation [15]. Serum levels of gastroenterological hormones which contain motilin (MOT), nitrogen oxide (NO), vasoactive intestinal peptide (VIP), and serotonin (5-HT) were measured as a secondary outcome.

### 2.8. Statistical Analysis

Dates were analyzed using SPSS software version 19.0 (SPSS Inc., Chicago, IL, USA). We described variables by using means and standard deviations for continuous variables and percentages for categorical variables. One-way ANOVA or Kruskal–Wallis test were used to analyze continuous variables. The primary comparison was performed between TEAS and control groups. The sham group was set in order to rule out a possible placebo effect. Accordingly, the group differences (TEAS versus control and TEAS versus sham) were analyzed using Bonferroni or Dunnett's T3 post hoc test and did not require the ANOVA omnibus test to be significant. Categorical data was analyzed using Pearson's chi-square test or Fisher's exact test. P<0.05 was considered significant.

## 3. Results

### 3.1. The Patients' Baseline Characteristics

**Figure 2:**
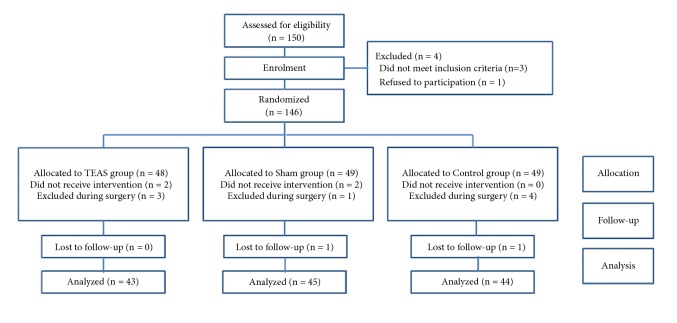
Consort flow diagram. The flowchart showing the study procedures and the number of patients. TEAS group, transcutaneous electrical acupoint stimulation; sham group, nonacupoint stimulation group; control group, no stimulation group.

A total of 150 patients were screened in this study as shown in Figure 2. Of these, 4 patients (2.7%) dropped out of the study, 3 patients did not meet the inclusion criteria, and 1 patient declined to receive TEAS. Finally, 146 patients participated in the study, 48 of whom were randomized to the TEAS group, 49 to the sham stimulation group, and 49 to the control group. 14 patients discontinued the study after randomization, 5 of whom were in the TEAS group, 4 in the sham group, and 5 in the control group. 4 patients did not receive TEAS because they were not able to tolerate the sensation of “De-Qi”, while 8 patients were excluded during surgery because of postpartum hemorrhage, which required stay in the intensive care unit (ICU) after surgery. 2 patients were lost to follow-up and we did not obtain the Barthel index. At last, 132 patients completed the study and their records were analyzed.

The baseline characteristics of the patients, including age, BMI, personal history of abdominal surgery, duration of operation, blood loss during surgery, total intraoperative infusion fluid during surgery, intraoperative medication, and postoperative analgesia, were similar between the TEAS group and the other two groups (Table 1).

### 3.2. Primary Outcomes

The time to passage of flatus was significantly shorter compared to control and sham groups (Table 2).

### 3.3. Second Outcomes

The time to defecation in TEAS group was significantly shorter than that in the control group, but there was no significant difference compared with sham group. The time to first oral liquid and solid intake and the postoperative hospital stay were significantly shorter in TEAS group compared to control and sham groups. The Barthel index at one week after surgery was significantly higher in TEAS group compared to the other two groups. The time to first mobilization after surgery and postoperative pain were similar between TEAS group and the other two groups (Table 2).

Regarding postoperative side-effects, only the incidence of abdominal distention was significantly decreased in TEAS group compared to control and sham groups. Besides, the severity of nausea and vomiting was significantly lower in TEAS group than that in control, but not sham group. The rates of nausea and vomiting as well as POI were similar between TEAS group and the other two groups (Table 2).

Measurement of serum levels of gastroenterological hormones before surgery demonstrated no significant differences between TEAS group and the other two groups. But the MOT level in TEAS group at 6 hours after surgery was significantly higher than that in control and sham groups, while the levels of VIP, NO, and 5-TH were still similar between TEAS group and the other two groups (Table 3).

## 4. Discussion

Pain, gastrointestinal dysfunction, and surgical immobility are the three major factors that generally delay the postoperative recovery of the patients. Therefore, pain relief, early recovery of gastrointestinal function, and ambulation are of great importance in postoperative care [16].

For thousands of years, acupuncture has been used to treat gastrointestinal symptoms in China. Also, earlier trials have demonstrated the beneficial effects of acupuncture on postoperative gastrointestinal function. Zhang and colleagues in his study confirmed that the first bowel movement and passage of flatus were shorter after colorectal surgery undergoing electroacupuncture at the acupoint ST36 [11]. Chen and colleagues showed that Evodia hot compress plus electroacupuncture treatment significantly decreased the time to defecation in patients undergoing abdominal surgery [17]. Similarly, SSMNg and colleagues also demonstrated that electroacupuncture shortened the time to defecation, length of hospital stay, and time to ambulation [18]. Despite these impressive data that favors the practice of postoperative acupuncture in colorectal surgery, the data supporting the role of acupuncture in cesarean section are quite limited. In this trial, we demonstrated that the time to first flatus passage time, oral liquid and normal diet intake, and the postoperative hospital stay were shorter in TEAS group compared to those in sham and control groups. Consistently, Barthel index at one week after surgery was higher and the incidence of abdominal distention was significantly lower in TAES group. These results indicated that TEAS is an effective technique in promoting postoperative gastrointestinal functional recovery in patients undergoing cesarean section.

Normally, early recovery of gastrointestinal function often results in the decreased incidence of POI, but the incidence of POI was not affected by TEAS in the current study. This might be because POI was not used as a primary outcome and the incidence of POI in patients undergoing cesarean section was relatively low. Thus, further trials with larger sample sizes are required to address this issue.

Although the cause of postoperative gastrointestinal dysfunction is multifactorial, recent studies suggested that intestinal inflammation triggered by surgical handling is one of the major causes of POI [19, 20]. Moreover, recent research has demonstrated that neurotransmitters including serotonin can play a significant role in the gastrointestinal physiology; these neurotransmitters can regulate gut blood flow, motility, nutrient absorption, gastrointestinal innate immune system, and the microbiome [21]. Also, previous studies have shown that electroacupuncture decreased the levels of inflammatory cytokines, such as IL-1*β*, IL-6, and TNF-*α*, in animal experiments and clinical trials [22–24]. This may in turn contribute to the beneficial effects of acupuncture in the treatment of postoperative gastrointestinal dysfunction. On the other hand, the enteric nervous system, humoral factors, and gastrointestinal hormones play an important role in regulating the gastrointestinal motility. To further validate whether the gastrointestinal hormones were involved in the acupuncture-induced regulation of gastrointestinal function still remains unknown. Interestingly, in the current study, we found that the serum level of MOT, a gastrointestinal hormone which can initiate phase III of gastric migration myoelectric complexes, demonstrated significantly higher values in TEAS group. However, the detailed mechanisms are still not clear, and hence further studies are required.

The acupoints used in this study was selected based on the theory of traditional Chinese medicine. The ST36 is considered as an acupoint on the Foot Yang Ming stomach meridian and very importantly regulates the functions of spleen and stomach [25]. Clinically, it is the most commonly used acupoint in treating gastrointestinal disorders [10, 16, 18, 26–28]. As indicated previously, electroacupuncture at ST36 has been shown to accelerate colonic transit and stimulate intestinal motility via parasympathetic and cholinergic pathways [29, 30]. SP6 acupoint on the spleen channel is also considered to be important and proved to be beneficial in treating various disorders, including gynecological, allergic, genitourinary, insomnia, psychosomatic, and immunological diseases and pain control [31]. Morgana and colleagues showed that manual acupuncture at SP6 acupoint inhibited vascular permeability, myeloperoxidase activity, and inflammatory cell infiltration in carrageenan-injected mice [32]. Xu and colleagues found that electroacupuncture at SP6 acupoint was effective in alleviating abdominal pain [33]. Considering these beneficial effects, acupoints ST36 and SP6 were used in the current trial. It is very likely that the therapeutic effects of TEAS at these two acupoints contributed to the TEAS-induced beneficial effects in our study.

An enhanced recovery program is one of the most popular developments in surgery recently. This multimodal program contains a number of perioperative elements, which in turn aim for a faster postoperative recovery and earlier discharge. For this purpose, several approaches have been proposed in an attempt to promote the return of gastrointestinal motility after cesarean section, which included gum chewing, early oral hydration, and ambulation [34]. Compared with these complex elements of a fast-track program, TEAS is much simpler and easier to implement, demonstrated less side-effects, and may still benefit the patients by accelerating the recovery, shortening convalescence, and ultimately improving the activities of daily living. However, further studies with comparisons between fast-track program and TEAS in patients undergoing cesarean section are required.

Our study had several limitations. Firstly, the study population represented a highly selected group of patients undergoing elective cesarean section without any complications. Patients with diabetes, high blood pressure, or other complications were excluded, and these patients were more likely to experience a delayed gastrointestinal functional recovery after surgery. Also, it is still unclear whether TEAS is beneficial in the recovery of gastrointestinal function in other complications. Secondly, the patients screened in the current study were from one hospital, and large multicentre clinical trials are still needed to confirm these findings.

## 5. Conclusion

Overall, the findings from this trial suggest that TEAS accelerated gastrointestinal functional recovery, reduced postoperative hospital length, and improved activities of daily living after cesarean section. The increased serum levels of gastroenterological hormones in TEAS group suggested that these beneficial effects were partially mediated by the regulation of gastroenterological hormone.

## Figures and Tables

**Table 1:** Demographic data and perioperative details.

	TEAS (n=43)	Control (n=44)	Sham (n=45)	P-value
Age (yr)	32.2 (3.5)	31.4 (4.7)	30.6 (3.9)	0.224
Body mass index (kg m^−2^)	27.6 (2.8)	28.8 (2.7)	27.7 (2.0)	0.063
Gestation age (weeks)	38.7 (1.1)	38.7 (1.1)	38.2 (1.1)	0.101
Previous abdominal surgery	29 (67.4)	25 (56.8)	24 (52.4)	0.348
Duration of surgery (min)	41.9 (10.3)	42.0 (8.9)	43.7 (7.7)	0.586
Blood loss during surgery (mL)	426.1 (122.2)	439.8 (106.5)	397.6 (111.5)	0.220
Total intraoperative infusion (mL)	1195.4 (113.3)	1195.5 (161.3)	1188.1 (145.2)	0.963
Intraoperative medication†				
Ondansetron	4 (9.3)	7 (15.9)	9 (20.0)	0.370
Dexamethasone	8 (18.6)	6 (13.6)	6 (13.3)	0.743
Ephedrine	6 (14.0)	12 (27.3)	10 (22.2)	0.309
Atropine	2 (4.7)	2 (4.5)	0 (0.0)	0.344
Postoperative analgesia ‡				
None	3 (7.0)	3 (6.8)	2 (4.4)	0.855
Patient-controlled epidural analgesia	31 (72.1)	32 (72.7)	30 (66.7)	0.789
Patient-controlled intravenous analgesia	9 (20.9)	9 (20.5)	13 (28.9)	0.573

Values are mean (SD) or number (proportion). †The conditions of nausea and vomiting during surgery were treated with ondansetron 8 mg. 10 mg dexamethasone was given intravenously when there is possible nerve injury during anesthesia. Hypotension (fall in systolic blood pressure (SBP)>20% from baseline) and bradycardia (heart rate (HR) <55 beats/min) were treated with 10 mg ephedrine and 0.5 mg atropine through intravenous bolus injection, respectively. ‡Epidural patient-controlled analgesia was established with 100 mL of 0.05mg mL^−1^ morphine plus 2mg mL^−1^ ropivacaine, programmed to deliver a 1 mL bolus with a lockout interval of 15 min and a background infusion of 2 mL h^−1^. Intravenous patient-controlled analgesia was established with 100 mL of 2 *μ*g mL^−1^ sufentanil, programmed to deliver a 1 mL bolus with a lockout interval of 15 min and a background infusion of 2 mL h^−1^.

**Table 2:** Outcome measurements in TEAS, control, and sham groups.

	TEAS (n=43)	Control (n=44)	Sham (n=45)	P-value	
				TEAS *vs* Control	TEAS *vs* Sham
Primary outcomes					
First flatus passage (h)	19.3 (10.0)	26.2 (12.8)	26.4 (10.1)	0.004	0.003
First defecation (h)	28.9 (25.2)	42.5 (30.8)	41.3 (29.4)	0.039	0.052
Oral liquid intake (h)	19.8 (5.2)	26.2 (8.6)	23.0 (3.0)	<0.001	0.019
Solid intake (h)	44.4 (12.4)	51.8 (13.8)	51.2 (15.1)	0.021	0.037
Second outcomes					
Early mobilization (h)	28.2 (6.3)	30.5 (7.6)	28.9 (2.6)	0.086	0.625
Postoperative hospital stay (h)	92.2 (16.1)	100.2 (15.7)	106.7 (12.6)	0.031	<0.001
Barthel index (score)	94.9 (4.9)	88.4 (9.2)	90.0 (9.5)	0.001	0.015
Incidence of nausea or vomiting	11 (30.6)	8 (18.2)	6 (13.3)	0.283	0.118
VDS for nausea and vomiting	0.1 (0.2)	0.3 (0.7)	0.2 (0.4)	0.048	0.274
Incidence of abdominal distention	9 (20.9)	21 (47.7)	19 (42.2)	0.013	0.040
Incidence of POI	3 (7.0)	1 (2.3)	3 (6.7)	0.360	1.000
NRS for pain at rest (score)					
6 h after surgery	1.7 (1.0)	1.6 (0.9)	1.6 (0.9)	0.833	0.612
24 h after surgery	1.4 (0.9)	1.6 (1.1)	1.6 (1.1)	0.470	0.473
48 h after surgery	0.9 (0.8)	1.1 (0.8)	0.7 (0.8)	0.868	0.986
NRS for pain with movements (score)					
6 h after surgery	3.0 (1.7)	2.9 (1.8)	3.2 (1.5)	0.711	0.688
24 h after surgery	1.8 (1.5)	2.1 (1.4)	2.2 (1.5)	0.445	0.233
48 h after surgery	1.2 (1.0)	1.1 (0.8)	1.2 (1.0)	0.279	0.340

Values are presented as mean (SD) or number (proportion). POI: postoperative ileus. VDS: verbal descriptive scale; no nausea and vomiting = VDS 0; mild: patient reports nausea but declines antiemetic treatment = VDS 1; moderate: patient reports nausea and accepts antiemetic treatment = VDS 2; severe: nausea with any emesis episode(retching or vomiting) = VDS 3. NRS: numeric rating scale; the NRS for pain is a unidimensional measure of pain intensity in adults; the scores range from 0 to 10; higher scores indicate greater pain intensity. Barthel index is a scoring technique that measures patient's performance in ten activities of daily life; the scores range from 0 to 100; for clinical evaluation, 76-100 points denote “good function”, 51-75 points denote “moderate disability”, and score under 50 denotes “severe disability”.

**Table 3:** Serum levels of gastroenterological hormones in TEAS, control, and sham groups.

	TNAS (n=43)	Control (n=44)	Sham (n=45)	P-value	
				TEAS *vs* Control	TEAS *vs* Sham
Before surgery					
MOT (ng mL^−1^)	27.1 (19.4)	35.9 (23.4)	27.1 (23.6)	0.165	0.998
VIP (ng Ml^−1^)	33.4 (29.2)	30.0 (19.4)	41.6 (33.1)	0.762	0.515
NO (ng mL^−1^)	712.6 (228.2)	737.8 (295.5)	746.8 (305.2)	0.713	0.686
5-HT (ng ML^−1^)	13.0 (2.8)	13.4 (3.4)	13.0 (4.6)	0.630	0.984
After surgery					
MOT (ng mL^−1^)	59.6 (24.8)	38.1 (20.2)	37.7 (25.4)	0.014	0.020
VIP (ng mL^−1^)	23.5 (17.2)	30.6 (22.2)	35.6 (30.0)	0.456	0.267
NO (ng mL^−1^)	801.3 (325.0)	839.3 (303.3)	802.6 (238.6)	0.647	0.989
5-HT (ng mL^−1^)	10.7 (2.5)	11.2 (3.0)	11.8 (3.4)	0.590	0.279

Values are presented as mean (SD). MOT: motilin; VIP: vasoactive intestinal peptide; NO: nitrogen oxide; 5-HT: serotonin.

## Data Availability

The data used to support the findings of this study are available from the corresponding author upon request.

## Abbreviations

TEAS: Transcutaneous electrical acupoint stimulation
POI: Postoperative ileus
NRS: Numeric rating scale
VDS: Verbal descriptive scale
MOT: Motilin
NO: Nitrogen oxide
VIP: Vasoactive intestinal peptide
5-HT: Serotonin
SD: Standard deviation
BMI: Body mass index.
